# Supplementary material for: Neural encoding of pain is robust within but unstable between individuals
Source: PLoS Biol. 2026 Aug 10;24(8):e3003948. doi: 10.1371/journal.pbio.3003948 (PMC13475984; doi:10.1371/journal.pbio.3003948)
Supplement: Supplementary file 1 [file pbio.3003948.s006.docx]

**Reply letter**

**Reviewer #1:**

This manuscript presents a rigorous investigation into the neural encoding of pain variability, distinguishing between intra-individual (within-person) and inter-individual (between-person) differences. The authors employ electroencephalography (EEG) and Bayesian multi-model regression to analyze responses to painful stimuli across a substantial cohort, including repeated measures and an independent replication dataset. A key finding is that neural patterns associated with intra-individual pain fluctuations demonstrate high robustness, being both repeatable over time and replicable across independent cohorts. In contrast, neural patterns linked to inter-individual pain differences are repeatable within the same cohort but fail to replicate in an independent one. These findings underscore the distinct nature of neural encoding for different sources of pain variability and highlight the potential utility of within-person approaches for developing clinically relevant pain biomarkers. The study's clear presentation and systematic approach to robustness assessment are commendable.

We thank the reviewer for their positive assessment of our manuscript.

1.) The analysis relies on a pre-defined set of traditional EEG features (N1, N2/P2 amplitudes, and power in alpha, beta, and gamma bands) extracted from specific electrodes and time/frequency windows. While these features are well-established, their selection is inherently hypothesis-driven and may not capture the full complexity of neural encoding present in the raw EEG data. Could the authors discuss the potential for bias introduced by this constrained feature selection? Specifically, would the application of data-driven methods, such as deep learning directly on raw or minimally processed EEG data, potentially reveal different or more comprehensive neural patterns, and how might such an approach complement or challenge the current findings?

We thank the reviewer for this thoughtful comment. The choice of the predefined set of EEG features was motivated by prior research demonstrating that these features are particularly relevant for characterizing pain-related neural activity. Moreover, they exhibit relatively high signal-to-noise ratios. Importantly, the features were defined in our preregistration before any analyses were conducted, thereby minimizing the risk of data-driven feature selection bias.

We fully agree that data-driven approaches, including deep learning models applied to raw or minimally processed EEG signals, may reveal additional neural patterns and improve predictive performance. We are currently pursuing this line of research. However, while such approaches may identify additional informative signal components, we feel that the current approach still allows for the central conclusion of the present study: namely, that neural patterns associated with intra-individual pain variability are stronger and more robust than those associated with inter-individual differences.

We now explicitly discuss the potential of data-driven modeling:

*“Importantly, our findings have to be interpreted in the context of our modeling choices. Specifically, we focused on a predefined set of established post-stimulus EEG features selected based on prior evidence. Consequently, other potentially informative signal components, including pre-stimulus neural activity and features derived using more data-driven approaches, were not considered. Future studies may reveal additional neural patterns and improve predictive performance by incorporating such information.”*

2.) The study utilizes laser-induced experimental pain, which, by its controlled nature, differs significantly from the multifaceted and often chronic pain experienced in real-life clinical settings. While experimental models offer valuable insights into fundamental mechanisms, the extent to which findings derived from acute, experimental noxious stimuli can be directly translated to the understanding, diagnosis, or management of clinical pain remains a critical question. Could the authors elaborate on the limitations of generalizing these findings to diverse clinical pain populations and discuss strategies for bridging this gap in future research?

We agree that our findings cannot be directly generalized to clinical pain conditions. Importantly, we do not suggest that the specific patterns of neural features identified as explaining variability in the perception of experimentally induced pain play a similar role in clinical or chronic pain. Rather, we propose that the broader principle emerging from our findings, i.e., that intra-individual variability in pain perception can be decoded more robustly than inter-individual variability, may extend to clinical pain populations.

We emphasize this now more explicitly in the discussion:

*“The extent to which the differential decodability of intra- and inter-individual pain variability generalizes to clinical pain populations remains an important question for future research. Nevertheless, the associations identified here may provide a foundation for future mechanistic studies and for testing whether similar principles govern pain variability in clinical populations.”*

3.) The current analysis primarily focuses on event-related EEG features, which reflect brain responses *following* stimulus presentation. However, pre-stimulus brain states, influenced by factors such as attention, expectation, and baseline cortical excitability, are known to significantly modulate pain perception. Did the authors consider the contribution of pre-stimulus neural activity to pain variability? Integrating such features could potentially offer a more comprehensive model of pain encoding and provide further insights into the observed intra- and inter-individual differences.

We agree that pre-stimulus neural activity can substantially influence pain perception and may provide additional insights into both intra- and inter-individual pain variability. However, in the present study, we deliberately focused on a predefined set of established pain-related EEG features that capture prominent post-stimulus responses and are expected to exhibit relatively high signal-to-noise ratios. To maximize the robustness and interpretability of our analyses, and consistent with our preregistration, pre-stimulus features were therefore not included. Investigating the contribution of pre-stimulus EEG activity to pain variability is an important direction for future research and is currently being pursued in our ongoing work.

We now mention the potential assessing pre-stimulus EEG signal in the discussion:

*“Consequently, other potentially informative signal components, including pre-stimulus neural activity and features derived using more data-driven approaches, were not considered. Future studies may reveal additional neural patterns and improve predictive performance by incorporating such information.”*

4.) The study's use of an independent internal cohort for replication is a strong methodological asset. To further strengthen the claims regarding the generalizability of the findings, particularly for the robust intra-individual patterns, have the authors explored the possibility of validating their findings using publicly available or collaboratively shared LEP datasets from other research laboratories? Such cross-laboratory validation would provide invaluable evidence for the broader applicability of the identified neural signatures, accounting for potential variations in experimental setups, participant demographics, and data acquisition protocols across different research sites.

We fully agree that validation in independent datasets collected by other research laboratories is critical for establishing the generalizability of neurophysiological pain markers. In fact, this was a central component of the present study. Specifically, we validated our findings in an independent LEP dataset collected by a separate research group in China, thereby testing the robustness of the identified patterns across laboratories, participant samples, and data acquisition settings. Notably, the intra-individual patterns identified in the original cohort replicated in this external dataset, whereas the inter-individual patterns did not. We agree that future validation in additional independent datasets would provide further evidence regarding the generalizability of these findings.

**Reviewer #2 [David M.A. Mehler]:**

The submitted manuscript sumarises in a concise and clear way a well-planne, pre-registered study that investigates a research question of high relevance. Pain so far relies on subjetive markers show show substantial inter-individual variability. How is this reflected in key neural correlates of pain? Work in this direction gives better insights into underlying neural mechanisms of pain and is is an important step towards developing reliable brain-based markers for potential clinical use. The submitted work provides a very transparent and clear design, the sequential Bayesian sampling plan introduces an innovative statistical framework to this field.

We thank the reviewer for their positive assessment of our manuscript.

A few aspects related to the sampling plan require some clarification/adjustment in the reporting:

1.) "Thus, the final sample size was n = 161 (82/80 female/male; ...) --> was it 162 or 161 participants?

Thank you for pointing out this inconsistency. The correct number is 81 male. We have corrected this in the manuscript

2.) In Bayesian frameworks, there is no concept of power, but sensitivity; please adjust this detail  (e.g. in lines 708, 711, 716 and 720).

We thank the reviewer for this comment. We agree that, in a Bayesian framework, the term "sensitivity" is more appropriate than "power." We have therefore revised the terminology throughout the manuscript accordingly:

*„Statistical sensitivity. Since our analyses are based on Bayesian multi-model regression and Bayesian linear mixed-effects models (LMEs), which are not currently supported by Bayes factor design analysis (BFDA)[57] tools, we performed an approximate statistical sensitivity analysis using a Bayesian correlation model as a proxy. Using the R package “BFDA” [58] with default priors and 10,000 simulations, we estimated statistical sensitivity for our sample size of n = 161 participants. Assuming a medium-sized correlation (R = 0.3) and BF_10_ thresholds of 1/3 and 3, the analysis yielded a statistical sensitivity of 94% with a false positive rate of 0.2%. Under more conservative thresholds (BF_10_ = 1/10 and 10), sensitivity was 86% with 0% false positives. These simulations serve as an approximate indication that the sample size allowed for a statistical sensitivity sufficient to detect medium-sized effects. While they do not fully reflect the complexity of the models, i.e. their hierarchical structure, they offer a reasonable estimate in the absence of validated Bayesian sensitivity estimation tools for regression and mixed-effects models.“*

3.) I could not find the BFDA simulation code or output on OSF; would it be possible to share these?

The BFDA has been performed very early during the project, and the code has not been stored, so it can unfortunately not be provided.

5.) After data exclusion, only 111 participants remained, hence substantially less sensitivity was achieved than planned based on the BFDA. This aspects seems not to be discussed? How does this affect individual results?

We agree that the final sample size of the replication dataset (n = 111) was smaller than the sample size considered in the approximate BFDA. However, the primary purpose of the replication dataset was not model development but independent validation. Specifically, model parameters were estimated in the internal dataset and subsequently used to generate predictions in the replication dataset. Thus, the replication analyses yielded informative evidence despite the reduced sample size. Evidence for an association between predicted and observed pain ratings was strong for intra-individual pain variability (BF > 10^28^), whereas evidence against such an association was at least moderate for inter-individual pain variability (BF < 1/3). Consistent with these Bayes factors, the corresponding models explained approximately 13% and 0% of the variance, respectively. Thus, although the reduced sample size lowered the sensitivity of the replication analyses relative to the original design assumptions, it did not hinder the accumulation of substantial evidence either for or against the respective effects.

6.) The abstract mentions N=162, and then an indepenendet cohort of N=111; was N=111 indendent? Lines 277 to 280 read as if these 111 participants were left after data exclusion of participants who did not show enough "performance" (i.e., trials that fulfill certain requirements). This would not be independent, but data selection. Presumably, this section (following lines 266) pertains the independent Chinese sample that is explained in more detail in lines 519-556? Please clarify.

We thank the reviewer for pointing out this potential ambiguity. The replication cohort (N = 111) was fully independent from the primary cohort and corresponds to the dataset described in the section “External replication data”. The reported sample size reflects the application of predefined data quality criteria, not participant performance. Specifically, participants with insufficient artifact-free trials were excluded. These quality control procedures were applied independently to both the internal and external datasets. We have revised the corresponding section to clarify that the final sample of 111 participants represents an independent replication cohort after quality-control exclusions:

*„Data selection criteria were applied analogously to the preregistered criteria used in the internal sample. Consequently, we included only higher-intensity trials, and excluded participants with fewer than 5 clean trials. This yielded a final replication sample of 111 individuals.”*

7.) Importantly, please indicate if and where this data selection criterion was part of the preregistration? This is a crucial point, as post-hoc analytical decisions on this level are considered problematic and pre-registration a major tool to provide transparency.

Data exclusion criteria defined for the internal sample are provided in the “Data exclusion” section in the preregistration. Specifically, we wrote in the preregistration: “The following exclusion criteria will be applied: 1. Single trials will be omitted from all analyses if (a) artifacts will be detected in the preprocessed EEG data during time periods used for baseline correction or the 1000 ms time interval following the laser stimulus. (b) no/an invalid pain rating (outside to the range of 0-100) occurred. 2. Participants will be excluded if < 5 trials (25%) remain per stimulation intensity after applying above criteria.”

The replication analyses were not part of the original preregistration, as the independent replication dataset became available only after the preregistration had been published. We included these analyses to extend the study and evaluate the robustness of our findings in an external cohort. We state this now more clearly in the manuscript:

*“The external dataset became available only after publication of the preregistration and was therefore not included in the preregistered analyses.”*

8.) The pre-registation otherwise provides many details and is hence a great example for transparency. Besides pre-registration, Registered Reports provide an even more robust way (e.g., <https://www.nature.com/articles/s41562-021-01193-7>), for instance, with respect to the possibility of publishing non-conclusive findings (<https://journals.plos.org/plosbiology/article?id=10.1371/journal.pbio.3000246>). This should be discussed.

We agree that Registered Reports are an important mechanism for promoting transparency and reproducibility. However, we respectfully feel that a discussion of publication formats and research workflows would extend beyond the scope of the present manuscript, which is focused on the scientific findings and their interpretation.

**Reviewer #3:**

The study addresses three clearly defined research questions, and the main conclusion is appropriately summarized in the title. Overall, the methodology appears suitable for investigating evoked pain responses. However, caution is warranted when extending these conclusions to the chronic pain population. In particular, the limited number of EEG electrodes used in the study may not support broad or categorical claims regarding the robustness of pain markers.

We thank the reviewer for their positive assessment of our manuscript.

I have only a few minor comments:

1.) Figure 2: Although the figure is clear for readers with expertise in EEG analysis, it may be helpful to explicitly state that, in panel B, each scalp topography corresponds to one of the highlighted squares in the time-frequency plot.

We thank the reviewer for pointing this out. We have now adapted the figure legend accordingly:

*„Scalp topographies illustrate the spatial distributions of relative power changes, averaged within the time-frequency windows indicated with dashed rectangles (from bottom to top: alpha, beta, gamma).”*

2.) Figure 3B: The intra-individual distribution of long-term pain appears narrower than that of short-term pain. Intuitively, I would have expected the opposite pattern. The authors may wish to comment on this observation and provide a possible explanation.

We agree that, in principle, day-to-day fluctuations in pain perception could exceed moment-to-moment fluctuations. However, substantial trial-to-trial variability is a well-established feature of pain perception and was therefore expected in our data. To characterize long-term (day-to-day) variability, we calculated session-level pain scores by averaging pain ratings across all trials within a session. Given the combination of substantial within-session variability in trial-level pain ratings and the bounded rating scale (0–100), we consider a narrower distribution of session-level pain scores plausible. Specifically, large moment-to-moment fluctuations can span much of the available scale, whereas averaging across trials attenuates these short-term fluctuations and yields session-level values that occupy a narrower range.

3.) Page 10 - Brain patterns of inter-individual variability of pain: Please consider including the equations used to calculate the reported measures, as this would improve clarity and reproducibility.

Thank you for this opportunity to clarify our methods. We now include mathematical definitions in the methods section:

*“To compute subject-level values (i.e., subject-specific averages of brain responses and pain ratings), we first averaged trial-level values separately for each of the two stimulus intensities and then averaged the resulting intensity-specific means. Formally,*

$\hat{x}_{i} = \frac{1}{2}\sum_{s=1}^{2} \frac{1}{N_{is}}\sum_{t=1}^{N_{is}} x_{ist}$*,*

*where x_ist_ denotes the trial-level value for individual i, stimulus intensity s, and trial t, and N_is_ denotes the number of available trials for individual i at stimulus intensity s.“*

4.) Discussion (lines 389-390): The statement that "These findings challenge the feasibility of developing robust EEG-based diagnostic markers…" may be overstated. I do not believe that evoked pain responses measured in healthy participants using only a few central electrodes can be directly compared with the complex neurophysiological alterations observed in chronic pain conditions. Chronic pain is associated with widespread changes in brain networks and cortical reorganization involving much larger brain regions. Furthermore, EEG power spectra and other electrophysiological indices are known to differ substantially between healthy individuals and patients with chronic pain.
I therefore suggest that the authors expand the discussion to address how chronic pain affects EEG activity and whether such changes could be assessed using the same approach employed in the present study. It would also be valuable to discuss whether the investigation of chronic pain mechanisms may require different experimental paradigms, broader spatial EEG coverage, or alternative analytical approaches.

We agree that the original phrasing may have been too strong and have revised the corresponding passage to adopt a more nuanced wording:

*“Given that our experimental paradigm provides a highly controlled setting for probing neural representations of pain, these findings suggest that developing robust EEG-based diagnostic markers for clinical pain conditions may be challenging. This interpretation is consistent with recent studies highlighting the limited consistency and generalizability of EEG biomarker candidates [Zebhauser et al. 2022, Bott et al. 2025].”*

**Reviewer #4:**

The article "Neural Encoding of Pain is Robust Within but Unstable Between Individuals" presents a study investigating how EEG responses to noxious laser stimulation relate to variability in pain ratings within and between individuals. In a large sample of healthy participants tested twice approximately four weeks apart, and with replication in an independent cohort, the authors use Bayesian multi-model regression to relate canonical laser-evoked EEG responses — N1, N2, P2, and induced alpha, beta, and gamma responses — to subjective pain ratings. The main finding is that inter-individual differences in pain ratings are primarily associated with the P2 response and are repeatable within the same cohort, but do not replicate in an independent cohort. In contrast, intra-individual fluctuations in pain ratings are associated with a broader set of EEG responses and show stronger robustness, being repeatable across sessions and replicable across cohorts. The authors conclude that EEG-based markers may be better suited to tracking within-person fluctuations in pain than to explaining stable differences in pain sensitivity between individuals. Overall, the manuscript is well written and methodologically sound. The large sample size, pre-registration, use of an external replication cohort, and explicit distinction between repeatability and replicability are major strengths.

We thank the reviewer for their positive assessment of our manuscript.

This said, some concerns should be addressed before publication:

1.) The choice of using four fixed intensities requires clearer justification. Were they included to increase the range of perceived pain, to reduce predictability, or for other reasons? The authors analyzed only trials corresponding to the two highest stimulation intensities. I understand that this was pre-registered and intended to optimise SNR. However, excluding half of the delivered stimuli may limit the interpretability and generalizability of the findings, especially because the study aims to characterize variability in pain perception.

We agree that the rationale for using different stimulus intensities requires clearer justification. The different stimulus intensities were primarily included to reduce stimulus predictability and to encourage the use of a broader range of the pain rating scale. We have now clarified this rationale in the results and methods sections.

Results: *“Stimuli were delivered at four different intensities to reduce stimulus predictability and to increase the range of pain intensity ratings.”*

Methods: *“Different stimulus intensities were included to reduce stimulus predictability and to encourage the use of a broader range of the pain rating scale.”*

Regarding the exclusion of the two lowest intensities, this preregistered decision was made to maximize signal-to-noise ratio and thereby improve the robustness of the analyses. We demonstrated the generalizability of results by assessing their replicability in an independent sample. Importantly, our study focused on variability in pain perception beyond that explained by stimulus intensity. Accordingly, analyses of intra-individual pain variability were based on residual pain ratings (i.e., deviations from intensity-specific means) rather than absolute pain ratings. These residual ratings still exhibited substantial variability, consistent with the well-established trial-to-trial fluctuations in pain perception. We explain this now more clearly in the results section of the manuscript:

*“To optimize the signal-to-noise ratio, we only included trials with stimuli of the two highest intensities. We considered the resulting reduction in stimulus intensity-related variability acceptable because our primary aim was to investigate variability in pain perception beyond that explained by stimulus intensity.”*

2.) As admitted by the authors, the study cannot disentangle neural encoding of pain from neural encoding of stimulus intensity or general nociceptive/salience-related processing. However, the interpretation of the results still seems to drift toward conclusions about "pain" and "pain encoding" more broadly. This interpretation should be more nuanced.

We thank the reviewer for raising this point. We would like to clarify that our analyses were specifically designed to separate stimulus intensity-independent from stimulus intensity-dependent variability in pain perception. For example, analyses of intra-individual pain variability were based on residual pain ratings (i.e., deviations from intensity-specific means) rather than absolute pain ratings. Consequently, the identified neural patterns reflect variability in perceived pain beyond that explained by differences in stimulus intensity. To avoid potential misunderstandings, we have revised several sections of the manuscript to more clearly describe this aspect of the analysis.

Abstract: *“Here, we used electroencephalography (EEG) in 161 healthy participants to re-investigate the neural patterns explaining intra- and inter-individual variations in the perception of brief painful stimuli independent of stimulus intensity.”*

Introduction: *“Using Bayesian multi-model regression, we related trial-level and session-level pain ratings to evoked (N1, N2, P2) and induced oscillatory (alpha, beta, gamma) responses, focusing on variability in pain perception beyond that attributable to stimulus intensity.”*

Results: *“To isolate variability beyond that explained by stimulus intensity, analyses were performed on intensity-adjusted brain responses and pain ratings.”*

Discussion*: “Specifically, we focused on variability that could not be explained by differences in stimulus intensity.”*

3.) The manuscipt should engage more explicitly with the well-known limitations that LEPs are not specific markers of pain perception. LEPs are strongly influenced by stimulus salience, arousal, attention, and the detection of sudden sensory events. This does not undermine the study, as the authors' main question concerns robustness of EEG-rating associations rather than pain specificity. However, it does affect the interpretation of the findings.

We thank the reviewer for this important comment. We agree that the present data do not allow conclusions regarding the specificity of the identified EEG patterns for pain. At the same time, our study did not aim to assess the specificity of these neural responses, and we do not make claims regarding pain specificity. Rather, our conclusions concern the robustness of the association between EEG features and pain ratings. To acknowledge this limitation more explicitly, we have expanded the discussion accordingly:

*“Our study has limitations, particularly regarding the specificity of the reported effects. First, it remains unclear whether the identified EEG patterns are specific to pain perception or generalize to other sensory modalities and perceptual processes. In particular, laser-evoked potentials and associated oscillatory responses are known to be influenced not only by pain perception but also by more general processes such as stimulus salience, arousal, and attention.”*
