# Supplementary material for: Neural encoding of pain is robust within but unstable between individuals
Source: PLoS Biol. 2026 Aug 10;24(8):e3003948. doi: 10.1371/journal.pbio.3003948 (PMC13475984; doi:10.1371/journal.pbio.3003948)
Supplement: Supplementary file 2 [file pbio.3003948.s007.docx]

**Reply letter**

**Editorial Suggestions**

Dear Dr Ploner,

Thank you for your patience while we considered your revised manuscript "Neural Encoding of Pain is Robust Within but Unstable Between Individuals" for consideration as a Research Article at PLOS Biology. Your revised study has now been evaluated by the PLOS Biology editors and the Academic Editor.

Based on these evaluations, we are pleased to offer you the opportunity to address the remaining editorial points in a revision that we anticipate should not take you very long. 

As discussed separately, these points include ensuring that all relevant code are made available and work as intended, and including a more explicit and balanced discussion of the implications of the findings for chronic pain.

All relevant code, including code for the Bayes Factor Design Analysis (BFDA), is now openly available on <https://osf.io/bs3yj>. Moreover, we have now included an additional paragraph in the discussion that provides a more explicit and balanced account of the implications of the findings for chronic pain.

We will then assess your revised manuscript and your response to the reviewers' comments with our Academic Editor aiming to avoid further rounds of peer-review, although we might need to consult with the reviewers, depending on the nature of the revisions. We also ask that you address some additional policy-related requests:

--------------------
IMPORTANT: Please make sure that your revision also addresses the following policy-related points:

**Ethics:

The Ethics statement needs to be a separate, independent (and the first) subheading in the Material & Methods section. Please move all of the information about ethical approvals to this new subheading. Please also indicate whether and how (written/oral) all participants gave consent to participate.

Done as suggested.

**Data:

Thank you for providing the raw EEG data on OSF. We have a few additional requests related to our data provision policy., as we require that the numerical data as they underly the figures be made available. Could you please add a file containing these (processed) data, either in the OSF repository or as a Supporting Information file "S1 Data" ("S1_Data.xlsx")? This applies to the following figure panels, which you can include in the same file using tabs:

3AB
4
5AB
S2AB
S3
S4
S5AB

We now provide the processed data in the OSF directory (https://osf.io/bs3yj/files/osfstorage)

In addition, please cite the location of these underlying data in all of the relevant figure and supplemental figure legends, e.g. “The data underlying this Figure can be found in S1 Data” or “The data underlying this Figure can be found in <https://doi.org/10.5281/zenodo.XXXXX>”.

We indicated the location of the underlying processed data in the figure legends: “The data underlying this Figure can be found at https://osf.io/bs3yj/files/osfstorage.”

Please also include and refer to the OSF Project DOIs in the Data Statement in the submission form.

Done as suggested.


**Code:

Thank you for uploading your code to OSF. Please make the code available prior to acceptance so that we can evaluate it, and include the DOI. Please note that the code should include a use license and a README file, and needs to include all of the custom code and scripts related to this study.

All code related to the study is now available at <https://osf.io/bs3yj/files/osfstorage>, including a README file and a license.

**Journal Requirements**

1) Thank you for stating "Code used for the current manuscript is publicly available at <https://osf.io/bs3yj>

This is asking for sign in details. Please amend this to a new link or provide further details to locate the code

The code is now openly available via the provided link.

2) Please refer to the following article for PLOS Biology's best-practice recommendations for data presentation: https://journals.plos.org/plosbiology/article?id=10.1371/journal.pbio.1002128.

Guidelines for statistical reporting, if relevant, may be found here: <https://journals.plos.org/plosbiology/s/best-practices-in-research-reporting#loc-statistical-reporting>.

We have checked this useful resource and noticed that our data presentation is in good agreement with these recommendations.
