# Supplementary material for: Neural encoding of pain is robust within but unstable between individuals
Source: PLoS Biol. 2026 Aug 10;24(8):e3003948. doi: 10.1371/journal.pbio.3003948 (PMC13475984; doi:10.1371/journal.pbio.3003948)
Supplement: Peer review history

# Editor report: Decision Letter 0

**DOI:** 10.1371/journal.pbio.3003948.r001

**Authors:** Taylor Hart, PhD

1 May 2026

Dear Dr Ploner,

Thank you for submitting your manuscript entitled "Neural Encoding of Pain is Robust Within but Unstable Between Individuals" for consideration as a Research Article by PLOS Biology.

Your manuscript has now been evaluated by the PLOS Biology editorial staff, as well as by an academic editor with relevant expertise, and I am writing to let you know that we would like to send your submission out for external peer review.

However, before we can send your manuscript to reviewers, we need you to complete your submission by providing the metadata that is required for full assessment. To this end, please login to Editorial Manager where you will find the paper in the 'Submissions Needing Revisions' folder on your homepage. Please click 'Revise Submission' from the Action Links and complete all additional questions in the submission questionnaire.

Once your full submission is complete, your paper will undergo a series of checks in preparation for peer review. After your manuscript has passed the checks it will be sent out for review. To provide the metadata for your submission, please Login to Editorial Manager (https://www.editorialmanager.com/pbiology) within two working days, i.e. by May 03 2026 11:59PM.

If your manuscript has been previously peer-reviewed at another journal, PLOS Biology is willing to work with those reviews in order to avoid re-starting the process. Submission of the previous reviews is entirely optional and our ability to use them effectively will depend on the willingness of the previous journal to confirm the content of the reports and share the reviewer identities. Please note that we reserve the right to invite additional reviewers if we consider that additional/independent reviewers are needed, although we aim to avoid this as far as possible. In our experience, working with previous reviews does save time.

If you would like us to consider previous reviewer reports, please edit your cover letter to let us know and include the name of the journal where the work was previously considered and the manuscript ID it was given. In addition, please upload a response to the reviews as a 'Prior Peer Review' file type, which should include the reports in full and a point-by-point reply detailing how you have or plan to address the reviewers' concerns.

During the process of completing your manuscript submission, you will be invited to opt-in to posting your pre-review manuscript as a bioRxiv preprint. Visit http://journals.plos.org/plosbiology/s/preprints for full details. If you consent to posting your current manuscript as a preprint, please upload a single Preprint PDF.

Feel free to email us at plosbiology@plos.org if you have any queries relating to your submission.

Kind regards,

Taylor

Taylor Hart, PhD,

Associate Editor

PLOS Biology

thart@plos.org

# Peer review: Decision Letter 1

**DOI:** 10.1371/journal.pbio.3003948.r002

**Authors:** Taylor Hart, PhD

8 Jun 2026

Dear Dr Ploner,

Thank you for your patience while your manuscript "Neural Encoding of Pain is Robust Within but Unstable Between Individuals" was peer-reviewed at PLOS Biology. It has now been evaluated by the PLOS Biology editors, an Academic Editor with relevant expertise, and by several independent reviewers.

In light of the reviews, which you will find at the end of this email, we would like to invite you to revise the work to thoroughly address the reviewers' reports.

As you will see, the reviewers reported that the study appears robust and suitably transparent. They have commented on several aspects of the study that would benefit from further strengthening, including the justifications for some of the methodological & analytical choices, the discussion of generalizability and limitations of the chosen approach, some possible additional analyses, and necessary clarifications. In your revision, you should fully address the reviewers' points.

Given the extent of revision needed, we cannot make a decision about publication until we have seen the revised manuscript and your response to the reviewers' comments. Your revised manuscript is likely to be sent for further evaluation by all or a subset of the reviewers.

In addition to these revisions, you will need to complete some formatting changes, which you will receive in a follow up email. A member of our team will be in touch with a set of requests shortly.

We expect to receive your revised manuscript within 3 months. Please email us (plosbiology@plos.org) if you have any questions or concerns, or would like to request an extension.

At this stage, your manuscript remains formally under active consideration at our journal; please notify us by email if you do not intend to submit a revision so that we may withdraw it.

**IMPORTANT - SUBMITTING YOUR REVISION**

Your revisions should address the specific points made by each reviewer. Please submit the following files along with your revised manuscript:

1. A 'Response to Reviewers' file - this should detail your responses to the editorial requests, present a point-by-point response to all of the reviewers' comments, and indicate the changes made to the manuscript.

*NOTE: In your point-by-point response to the reviewers, please provide the full context of each review. Do not selectively quote paragraphs or sentences to reply to. The entire set of reviewer comments should be present in full and each specific point should be responded to individually, point by point.

You should also cite any additional relevant literature that has been published since the original submission and mention any additional citations in your response.

2. In addition to a clean copy of the manuscript, please also upload a 'track-changes' version of your manuscript that specifies the edits made. This should be uploaded as a "Revised Article with Changes Highlighted" file type.

*Re-submission Checklist*

When you are ready to resubmit your revised manuscript, please refer to this re-submission checklist: https://plos.io/Biology_Checklist

To submit a revised version of your manuscript, please go to https://www.editorialmanager.com/pbiology/ and log in as an Author. Click the link labelled 'Submissions Needing Revision' where you will find your submission record.

Please make sure to read the following important policies and guidelines while preparing your revision:

*Published Peer Review*

Please note while forming your response, if your article is accepted, you may have the opportunity to make the peer review history publicly available. The record will include editor decision letters (with reviews) and your responses to reviewer comments. If eligible, we will contact you to opt in or out. Please see here for more details:

https://blogs.plos.org/plos/2019/05/plos-journals-now-open-for-published-peer-review/

*PLOS Data Policy*

Please note that as a condition of publication PLOS' data policy (http://journals.plos.org/plosbiology/s/data-availability) requires that you make available all data used to draw the conclusions arrived at in your manuscript. If you have not already done so, you must include any data used in your manuscript either in appropriate repositories, within the body of the manuscript, or as supporting information (N.B. this includes any numerical values that were used to generate graphs, histograms etc.). For an example see here: http://www.plosbiology.org/article/info%3Adoi%2F10.1371%2Fjournal.pbio.1001908#s5

*Blot and Gel Data Policy*

We require the original, uncropped and minimally adjusted images supporting all blot and gel results reported in an article's figures or Supporting Information files. We will require these files before a manuscript can be accepted so please prepare them now, if you have not already uploaded them. Please carefully read our guidelines for how to prepare and upload this data: https://journals.plos.org/plosbiology/s/figures#loc-blot-and-gel-reporting-requirements

*Protocols deposition*

To enhance the reproducibility of your results, we recommend that if applicable you deposit your laboratory protocols in protocols.io, where a protocol can be assigned its own identifier (DOI) such that it can be cited independently in the future. Additionally, PLOS ONE offers an option for publishing peer-reviewed Lab Protocol articles, which describe protocols hosted on protocols.io. Read more information on sharing protocols at https://plos.org/protocols?utm_medium=editorial-email&utm_source=authorletters&utm_campaign=protocols

Thank you again for your submission to our journal. We hope that our editorial process has been constructive thus far, and we welcome your feedback at any time. Please don't hesitate to contact us if you have any questions or comments.

Sincerely,

Taylor

Taylor Hart, PhD,

Associate Editor

PLOS Biology

thart@plos.org

------------------------------------

REVIEWS:

Reviewer #1: This manuscript presents a rigorous investigation into the neural encoding of pain variability, distinguishing between intra-individual (within-person) and inter-individual (between-person) differences. The authors employ electroencephalography (EEG) and Bayesian multi-model regression to analyze responses to painful stimuli across a substantial cohort, including repeated measures and an independent replication dataset. A key finding is that neural patterns associated with intra-individual pain fluctuations demonstrate high robustness, being both repeatable over time and replicable across independent cohorts. In contrast, neural patterns linked to inter-individual pain differences are repeatable within the same cohort but fail to replicate in an independent one. These findings underscore the distinct nature of neural encoding for different sources of pain variability and highlight the potential utility of within-person approaches for developing clinically relevant pain biomarkers. The study's clear presentation and systematic approach to robustness assessment are commendable.

1. The analysis relies on a pre-defined set of traditional EEG features (N1, N2/P2 amplitudes, and power in alpha, beta, and gamma bands) extracted from specific electrodes and time/frequency windows. While these features are well-established, their selection is inherently hypothesis-driven and may not capture the full complexity of neural encoding present in the raw EEG data. Could the authors discuss the potential for bias introduced by this constrained feature selection? Specifically, would the application of data-driven methods, such as deep learning directly on raw or minimally processed EEG data, potentially reveal different or more comprehensive neural patterns, and how might such an approach complement or challenge the current findings?

2. The study utilizes laser-induced experimental pain, which, by its controlled nature, differs significantly from the multifaceted and often chronic pain experienced in real-life clinical settings. While experimental models offer valuable insights into fundamental mechanisms, the extent to which findings derived from acute, experimental noxious stimuli can be directly translated to the understanding, diagnosis, or management of clinical pain remains a critical question. Could the authors elaborate on the limitations of generalizing these findings to diverse clinical pain populations and discuss strategies for bridging this gap in future research?

3. The current analysis primarily focuses on event-related EEG features, which reflect brain responses *following* stimulus presentation. However, pre-stimulus brain states, influenced by factors such as attention, expectation, and baseline cortical excitability, are known to significantly modulate pain perception. Did the authors consider the contribution of pre-stimulus neural activity to pain variability? Integrating such features could potentially offer a more comprehensive model of pain encoding and provide further insights into the observed intra- and inter-individual differences.

4. The study's use of an independent internal cohort for replication is a strong methodological asset. To further strengthen the claims regarding the generalizability of the findings, particularly for the robust intra-individual patterns, have the authors explored the possibility of validating their findings using publicly available or collaboratively shared LEP datasets from other research laboratories? Such cross-laboratory validation would provide invaluable evidence for the broader applicability of the identified neural signatures, accounting for potential variations in experimental setups, participant demographics, and data acquisition protocols across different research sites.

Reviewer #2 [David M.A. Mehler]: The submitted manuscript sumarises in a concise and clear way a well-planne, pre-registered study that investigates a research question of high relevance. Pain so far relies on subjetive markers show show substantial inter-individual variability. How is this reflected in key neural correlates of pain? Work in this direction gives better insights into underlying neural mechanisms of pain and is is an important step towards developing reliable brain-based markers for potential clinical use. The submitted work provides a very transparent and clear design, the sequential Bayesian sampling plan introduces an innovative statistical framework to this field.

A few aspects related to the sampling plan require some clarification/adjustment in the reporting:

1. "Thus, the final sample size was n = 161 (82/80 female/male; ...) --> was it 162 or 161 participants?

2. In Bayesian frameworks, there is no concept of power, but sensitivity; please adjust this detail (e.g. in lines 708, 711, 716 and 720).

3. I could not find the BFDA simulation code or output on OSF; would it be possible to share these?

5. After data exclusion, only 111 participants remained, hence substantially less sensitivity was achieved than planned based on the BFDA. This aspects seems not to be discussed? How does this affect individual results?

6. The abstract mentions N=162, and then an indepenendet cohort of N=111; was N=111 indendent? Lines 277 to 280 read as if these 111 participants were left after data exclusion of participants who did not show enough "performance" (i.e., trials that fulfill certain requirements). This would not be independent, but data selection. Presumably, this section (following lines 266) pertains the independent Chinese sample that is explained in more detail in lines 519-556? Please clarify.

7. Importantly, please indicate if and where this data selection criterion was part of the preregistration? This is a crucial point, as post-hoc analytical decisions on this level are considered problematic and pre-registration a major tool to provide transparency.

8. The pre-registation otherwise provides many details and is hence a great example for transparency. Besides pre-registration, Registered Reports provide an even more robust way (e.g., https://www.nature.com/articles/s41562-021-01193-7), for instance, with respect to the possibility of publishing non-conclusive findings (https://journals.plos.org/plosbiology/article?id=10.1371/journal.pbio.3000246). This should be discussed.

Reviewer #3: The study addresses three clearly defined research questions, and the main conclusion is appropriately summarized in the title.

Overall, the methodology appears suitable for investigating evoked pain responses. However, caution is warranted when extending these conclusions to the chronic pain population. In particular, the limited number of EEG electrodes used in the study may not support broad or categorical claims regarding the robustness of pain markers.

I have only a few minor comments:

1.Figure 2

Although the figure is clear for readers with expertise in EEG analysis, it may be helpful to explicitly state that, in panel B, each scalp topography corresponds to one of the highlighted squares in the time-frequency plot.

2.Figure 3B

The intra-individual distribution of long-term pain appears narrower than that of short-term pain. Intuitively, I would have expected the opposite pattern. The authors may wish to comment on this observation and provide a possible explanation.

3.Page 10 - Brain patterns of inter-individual variability of pain

Please consider including the equations used to calculate the reported measures, as this would improve clarity and reproducibility.

4.Discussion (lines 389-390)

The statement that "These findings challenge the feasibility of developing robust EEG-based diagnostic markers…" may be overstated. I do not believe that evoked pain responses measured in healthy participants using only a few central electrodes can be directly compared with the complex neurophysiological alterations observed in chronic pain conditions.

Chronic pain is associated with widespread changes in brain networks and cortical reorganization involving much larger brain regions. Furthermore, EEG power spectra and other electrophysiological indices are known to differ substantially between healthy individuals and patients with chronic pain.

I therefore suggest that the authors expand the discussion to address how chronic pain affects EEG activity and whether such changes could be assessed using the same approach employed in the present study. It would also be valuable to discuss whether the investigation of chronic pain mechanisms may require different experimental paradigms, broader spatial EEG coverage, or alternative analytical approaches.

Reviewer #4: The article "Neural Encoding of Pain is Robust Within but Unstable Between Individuals" presents a study investigating how EEG responses to noxious laser stimulation relate to variability in pain ratings within and between individuals. In a large sample of healthy participants tested twice approximately four weeks apart, and with replication in an independent cohort, the authors use Bayesian multi-model regression to relate canonical laser-evoked EEG responses — N1, N2, P2, and induced alpha, beta, and gamma responses — to subjective pain ratings. The main finding is that inter-individual differences in pain ratings are primarily associated with the P2 response and are repeatable within the same cohort, but do not replicate in an independent cohort. In contrast, intra-individual fluctuations in pain ratings are associated with a broader set of EEG responses and show stronger robustness, being repeatable across sessions and replicable across cohorts. The authors conclude that EEG-based markers may be better suited to tracking within-person fluctuations in pain than to explaining stable differences in pain sensitivity between individuals.

Overall, the manuscript is well written and methodologically sound. The large sample size, pre-registration, use of an external replication cohort, and explicit distinction between repeatability and replicability are major strengths.

This said, some concerns should be addressed before publication:

1)The choice of using four fixed intensities requires clearer justification. Were they included to increase the range of perceived pain, to reduce predictability, or for other reasons? The authors analyzed only trials corresponding to the two highest stimulation intensities. I understand that this was pre-registered and intended to optimise SNR. However, excluding half of the delivered stimuli may limit the interpretability and generalizability of the findings, especially because the study aims to characterize variability in pain perception.

2)As admitted by the authors, the study cannot disentangle neural encoding of pain from neural encoding of stimulus intensity or general nociceptive/salience-related processing. However, the interpretation of the results still seems to drift toward conclusions about "pain" and "pain encoding" more broadly. This interpretation should be more nuanced.

3)The manuscipt should engage more explicitly with the well-known limitations that LEPs are not specific markers of pain perception. LEPs are strongly influenced by stimulus salience, arousal, attention, and the detection of sudden sensory events. This does not undermine the study, as the authors' main question concerns robustness of EEG-rating associations rather than pain specificity. However, it does affect the interpretation of the findings.

# Author comment: Author response to Decision Letter 2

**DOI:** 10.1371/journal.pbio.3003948.r003

16 Jun 2026

Attachment Submitted filename: reply_letter.docx

# Editor report: Decision Letter 2

**DOI:** 10.1371/journal.pbio.3003948.r004

**Authors:** Taylor Hart, PhD

6 Jul 2026

Dear Dr Ploner,

Thank you for your patience while we considered your revised manuscript "Neural Encoding of Pain is Robust Within but Unstable Between Individuals" for consideration as a Research Article at PLOS Biology. Your revised study has now been evaluated by the PLOS Biology editors and the Academic Editor.

Based on these evaluations, we are pleased to offer you the opportunity to address the remaining editorial points in a revision that we anticipate should not take you very long.

As discussed separately, these points include ensuring that all relevant code are made available and work as intended, and including a more explicit and balanced discussion of the implications of the findings for chronic pain.

We will then assess your revised manuscript and your response to the reviewers' comments with our Academic Editor aiming to avoid further rounds of peer-review, although we might need to consult with the reviewers, depending on the nature of the revisions. We also ask that you address some additional policy-related requests:

--------------------

IMPORTANT: Please make sure that your revision also addresses the following policy-related points:

**Ethics:

The Ethics statement needs to be a separate, independent (and the first) subheading in the Material & Methods section. Please move all of the information about ethical approvals to this new subheading. Please also indicate whether and how (written/oral) all participants gave consent to participate.

**Data:

Thank you for providing the raw EEG data on OSF. We have a few additional requests related to our data provision policy., as we require that the numerical data as they underly the figures be made available. Could you please add a file containing these (processed) data, either in the OSF repository or as a Supporting Information file "S1 Data" ("S1_Data.xlsx")? This applies to the following figure panels, which you can include in the same file using tabs:

3AB

4

5AB

S2AB

S3

S4

S5AB

In addition, please cite the location of these underlying data in all of the relevant figure and supplemental figure legends, e.g. “The data underlying this Figure can be found in S1 Data” or “The data underlying this Figure can be found in https://doi.org/10.5281/zenodo.XXXXX”

Please also include and refer to the OSF Project DOIs in the Data Statement in the submission form.

**Code:

Thank you for uploading your code to OSF. Please make the code available prior to acceptance so that we can evaluate it, and include the DOI. Please note that the code should include a use license and a README file, and needs to include all of the custom code and scripts related to this study.

--------------------

In addition to these revisions, you may need to complete some formatting changes, which you will receive in a follow up email. A member of our team will be in touch with a set of requests shortly. If you do not receive a separate email within a few days, please assume that checks have been completed, and no additional changes are required.

We expect to receive your revised manuscript within two weeks. Please email us (plosbiology@plos.org) if you have any questions or concerns, or would like to request an extension.

At this stage, your manuscript remains formally under active consideration at our journal; please notify us by email if you do not intend to submit a revision so that we withdraw the manuscript.

**IMPORTANT - SUBMITTING YOUR REVISION**

Your revisions should address the specific editorial points. Please submit the following files along with your revised manuscript:

1. A 'Response to Reviewers' file - this should detail your responses to the editorial request, and indicate the changes made to the manuscript.

*NOTE: In your point-by-point response to the reviewers, please provide the full context of each review. Do not selectively quote paragraphs or sentences to reply to. The entire set of reviewer comments should be present in full and each specific point should be responded to individually.

You should also cite any additional relevant literature that has been published since the original submission and mention any additional citations in your response.

2. In addition to a clean copy of the manuscript, please also upload a 'track-changes' version of your manuscript that specifies the edits made. This should be uploaded as a "Revised Article with Changes Highlighted " file type.

*Resubmission Checklist*

When you are ready to resubmit your revised manuscript, please refer to this resubmission checklist: https://plos.io/Biology_Checklist

To submit a revised version of your manuscript, please go to https://www.editorialmanager.com/pbiology/ and log in as an Author. Click the link labelled 'Submissions Needing Revision' where you will find your submission record.

Please make sure to read the following important policies and guidelines while preparing your revision:

*Published Peer Review*

Please note while forming your response, if your article is accepted, you may have the opportunity to make the peer review history publicly available. The record will include editor decision letters (with reviews) and your responses to reviewer comments. If eligible, we will contact you to opt in or out. Please see here for more details:

https://blogs.plos.org/plos/2019/05/plos-journals-now-open-for-published-peer-review/

*PLOS Data Policy*

Please note that as a condition of publication PLOS' data policy (http://journals.plos.org/plosbiology/s/data-availability) requires that you make available all data used to draw the conclusions arrived at in your manuscript. If you have not already done so, you must include any data used in your manuscript either in appropriate repositories, within the body of the manuscript, or as supporting information (N.B. this includes any numerical values that were used to generate graphs, histograms etc.). For an example see here: http://www.plosbiology.org/article/info%3Adoi%2F10.1371%2Fjournal.pbio.1001908#s5

*Blot and Gel Data Policy*

We require the original, uncropped and minimally adjusted images supporting all blot and gel results reported in an article's figures or Supporting Information files. We will require these files before a manuscript can be accepted so please prepare them now, if you have not already uploaded them. Please carefully read our guidelines for how to prepare and upload this data: https://journals.plos.org/plosbiology/s/figures#loc-blot-and-gel-reporting-requirements

*Protocols deposition*

To enhance the reproducibility of your results, we recommend that if applicable you deposit your laboratory protocols in protocols.io, where a protocol can be assigned its own identifier (DOI) such that it can be cited independently in the future. Additionally, PLOS ONE offers an option for publishing peer-reviewed Lab Protocol articles, which describe protocols hosted on protocols.io. Read more information on sharing protocols at https://plos.org/protocols?utm_medium=editorial-email&utm_source=authorletters&utm_campaign=protocols

Thank you again for your submission to our journal. We hope that our editorial process has been constructive thus far, and we welcome your feedback at any time. Please don't hesitate to contact us if you have any questions or comments.

Sincerely,

Taylor

Taylor Hart, PhD,

Associate Editor

PLOS Biology

thart@plos.org

# Author comment: Author response to Decision Letter 3

**DOI:** 10.1371/journal.pbio.3003948.r005

21 Jul 2026

Attachment Submitted filename: varPain_reply_letter_260713.docx

# Editor report: Decision Letter 3

**DOI:** 10.1371/journal.pbio.3003948.r006

**Authors:** Taylor Hart, PhD

29 Jul 2026

Dear Dr Ploner,

Thank you for the submission of your revised Research Article "Neural Encoding of Pain is Robust Within but Unstable Between Individuals" for publication in PLOS Biology. On behalf of my colleagues and the Academic Editor, Choong-Wan Woo, I am pleased to say that we can in principle accept your manuscript for publication, provided you address any remaining formatting and reporting issues. These will be detailed in an email you should receive within 2-3 business days from our colleagues in the journal operations team; no action is required from you until then. Please note that we will not be able to formally accept your manuscript and schedule it for publication until you have completed any requested changes.

Please take a minute to log into Editorial Manager at http://www.editorialmanager.com/pbiology/, click the "Update My Information" link at the top of the page, and update your user information to ensure an efficient production process.

PRESS

We frequently collaborate with press offices. If your institution or institutions have a press office, please notify them about your upcoming paper at this point, to enable them to help maximise its impact. If the press office is planning to promote your findings, we would be grateful if they could coordinate with biologypress@plos.org. If you have previously opted in to the early version process, we ask that you notify us immediately of any press plans so that we may opt out on your behalf.

We also ask that you take this opportunity to read our Embargo Policy regarding the discussion, promotion and media coverage of work that is yet to be published by PLOS. As your manuscript is not yet published, it is bound by the conditions of our Embargo Policy. Please be aware that this policy is in place both to ensure that any press coverage of your article is fully substantiated and to provide a direct link between such coverage and the published work. For full details of our Embargo Policy, please visit http://www.plos.org/about/media-inquiries/embargo-policy/.

Thank you again for choosing PLOS Biology for publication and supporting Open Access publishing. We look forward to publishing your study.

Sincerely,

Taylor

Taylor Hart, PhD,

Associate Editor

PLOS Biology

thart@plos.org
